# Inhibitory Efficacy of *Arthrospira platensis* Extracts on Skin Pathogenic Bacteria and Skin Cancer Cells

**DOI:** 10.3390/biology14050502

**Published:** 2025-05-05

**Authors:** Ranchana Rungjiraphirat, Nitsanat Cheepchirasuk, Sureeporn Suriyaprom, Yingmanee Tragoolpua

**Affiliations:** 1 Department of Biology, Faculty of Science, Chiang Mai University, Chiang Mai 50200, Thailand; ranchana_rung@cmu.ac.th (R.R.); nitsanat_cheep@cmu.ac.th (N.C.); sureeporn.suriyaprom@cmu.ac.th (S.S.); 2 Master of Science Program in Applied Microbiology (International Program), Faculty of Science, Chiang Mai University, Chiang Mai 50200, Thailand; 3 Office of Research Administration, Chiang Mai University, Chiang Mai 50200, Thailand; 4 Natural Extracts and Innovative Products for Alternative Healthcare Research Group, Faculty of Science, Chiang Mai University, Chiang Mai 50200, Thailand

**Keywords:** antibacterial, anticancer, antioxidant, *Arthrospira platensis*, skin pathogenic bacteria, spirulina

## Abstract

*Arthrospira platensis* (spirulina) is a cyanobacterium containing various phytochemical compounds associated with various antioxidant, antimicrobial, antiviral, anticancer, anti-inflammatory, and immune-promoting properties. The efficacy of ethanolic and methanolic crude extracts of *A. platensis* regarding antibacterial, antioxidant, and anticancer effects was determined in this study. The ethanolic extract showed the highest antioxidant activity by 8.96 ± 0.84 mg gallic acid equivalent per gram of extract (mg GAE/g extract), 53.03 ± 4.21 mg trolox equivalent antioxidant capacity per gram of extract (mg TEAC/g extract), and 48.06 ± 0.78 mg TEAC/g extract as determined by DPPH, ABTS, FRAP assays, respectively. Moreover, the ethanolic extract showed the highest total phenolic and flavonoid compound contents by 38.79 ± 1.61 mg GAE/g extract and 27.50 ± 0.53 mg of quercetin equivalent per gram of extract (mg QE/g extract). Gallic acid and quercetin in the extracts were also determined by HPLC. The antibacterial activity was evaluated by agar well diffusion and broth dilution methods on skin pathogenic bacteria, including *Staphylococcus aureus*, *Staphylococcus epidermidis*, methicillin-resistant *S. aureus* (MRSA), *Micrococcus luteus*, *Pseudomonas aeruginosa*, and *Cutibacterium acnes*. The inhibition zone of *A. platensis* extracts ranges from 9.67 ± 0.58 to 12.50 ± 0.50 mm. In addition, MIC and MBC values ranged from 31.25 to 125 mg/mL. The inhibition of biofilm formation and biofilm eradication by *A. platensis* ethanolic extract was 87.18% and 99.77%, as determined by the crystal violet staining assay. Furthermore, the anticancer activity of *A. platensis* on A375 human melanoma cells was examined. The ethanolic and methanolic extracts induced DNA apoptosis through both intrinsic and extrinsic pathways by upregulating the expression of caspase-3, caspase-8, and caspase-9. These findings suggested that *A. platensis* demonstrated promising antioxidant, antibacterial, and anticancer activities, emphasizing its potential as a natural therapeutic agent for the management of pathological conditions.

## Simple Summary

*Arthrospira platensis* is a filamentous oxygenic photosynthetic cyanobacteria with high nutritional value. It contains numerous essential compounds, including proteins, carbohydrates, vitamins, lipids, minerals, and pigments. These components contribute to its diverse biological properties. Our study examined the biological potential of ethanolic and methanolic crude extracts of *A. platensis*, focusing on its antioxidant, antibacterial, and anticancer properties. The antioxidant activity of the *A. platensis* extracts was assessed by DPPH, ABTS, and FRAP assays, as well as the determination of total phenolic and flavonoid compound contents. The antibacterial effect was investigated against skin pathogenic bacteria using agar well diffusion and broth dilution methods, along with biofilm inhibition and eradication. The anticancer activity was assessed by the cytotoxic effect, apoptosis induction, and gene expression in A375 human melanoma cells. The ethanolic extracts of *A. platensis* illustrated strong antioxidant and antibacterial activities. Conversely, the methanolic extract effectively induces apoptosis in A375 cells through both extrinsic and intrinsic pathways. This finding indicated that the ethanolic and methanolic extracts of *A. platensis* possess potential antioxidant, antibacterial, and anticancer properties, making them a promising agent for treating skin bacterial infection and skin cancer.

## 1. Introduction

Skin is the largest organ in the body, representing 15–20% of total body weight. It has various important functions, including preventing dehydration, body temperature regulation, sensory reception, maintaining fluid balance, vitamin and hormone production, and self-healing properties [1]. Moreover, it is responsible as a protective barrier, which protects the internal organs from the external environment, such as sunlight, ultraviolet (UV) radiation, chemical and physical injuries, pollutants, and microorganism infection [2]. The skin undergoes physiological changes that result in considerable modifications in the microbiota [3]. Furthermore, endogenous and exogenous factors such as temperature, humidity, salinity, age, sex, immune system, and daily activities also affect the growth of skin bacteria [4]. Consequently, an imbalance of growth between commensal bacteria and pathogenic bacteria can cause skin diseases. Skin and subcutaneous diseases emerged as leading causes of illness, experiencing a 46.8% increase between 1990 and 2017, and ranking as the fourth most prevalent disease globally [5]. Among these conditions, dermatitis is the highest global burden of skin disease and can be found in all ages.

Skin cancer is one of the skin diseases apart from bacterial infection. It is the uncontrolled proliferation of abnormal cells that then invade and damage surrounding normal body tissues. There are various types of skin cancer, with non-melanoma being the most common, including basal cell carcinoma (BCC) and squamous cell carcinoma (SCC). Malignant melanoma is the most severe type because it can spread through the bloodstream and other organs, such as lymph nodes [6]. The incidence rates of melanoma and non-melanoma skin cancers are on the rise in the U.S. Each year, approximately 5.4 million new cases of BCC and SCC are diagnosed [7]. Additionally, as of 2021, around 1.5 million individuals were living with skin melanoma. In 2024, there were 100,640 new cases of malignant melanoma reported, with an estimated 8290 deaths, ranking it as the fifth most common cancer [8]. Skin cancer can be stimulated by many factors such as chronic sun or UV exposure, chemical exposure, and individual skin color. Furthermore, skin cancer can be caused by free radicals, which are produced by irregular metabolic mechanisms in the body. These free radicals interact with cellular biomolecules such as nucleic acids, proteins, carbohydrates, and lipids. Accordingly, it leads to many problems, including cell damage and impairment of biological structures and function of tissues, and is associated with the development of various diseases, such as immunodeficiency and cancer [9]. Skin cancer can be treated by surgery, radiation, chemotherapy, cryotherapy, and immunotherapy. However, these treatments have side effects and downstream complications in patients, especially for the elderly or individuals with congenital diseases [10].

*Arthrospira platensis*, commonly referred to “Spirulina”, is ubiquitous oxygenic photosynthetic cyanobacteria that is found in freshwater and marine environments. It is an important source of protein, comprising approximately 60–70% of both essential and non-essential amino acids. Moreover, it has another nutritional value, such as lipids, carbohydrates, vitamins, minerals, and pigments [11,12]. *A. platensis* is promising microalgae that has been studied for antioxidant, antimicrobial, antiviral, anti-inflammation, and anticancer properties [13]. Notably, the U.S. FDA has certified *A. platensis* as Generally Recognized as Safe (GRAS), and the WHO has recommended it as one of the superfoods. Additionally, NASA has selected *A. platensis* as a nutritional supplement for astronauts on space mission [14]. As a result, it is extensively used in numerous industries, such as dietary supplements, animal feeds, cosmetics, natural dyes, pharmaceuticals, and nutraceutical products [15].

Currently, antibiotics and chemicals are mostly used to treat bacterial infections and skin cancer. However, synthetic medications often have side effects on patients and can contribute to antimicrobial resistance. Consequently, a new approach using *A. platensis* extracts for treating bacterial infection and cancer cells has been investigated. This research aims to examine the biological properties of *A. platensis* crude extracts, including their antioxidant, antibacterial, and anticancer activities as well as their total phenolic and flavonoid compound contents.

## 2. Materials and Methods

### 2.1. Bacterial Strains

The skin pathogenic bacteria, including *Staphylococcus aureus* ATCC 25923, *Staphylococcus epidermidis* ATCC 14990, *Pseudomonas aeruginosa* ATCC 27853, *Micrococcus luteus*, and *Cutibacterium acnes* DMST 14,916, were obtained from SCB2711 Microbiology Laboratory, Department of Biology, Faculty of Science, Chiang Mai University, Thailand. The methicillin-resistant *S. aureus* (MRSA) 49 clinical isolate was obtained from the Clinical Microbiology, Department of Medical Technology, Faculty of Associated Medical Sciences, Chiang Mai University, Thailand.

### 2.2. Chemicals and Reagents

Mueller Hinton broth (MHB) and Brain Heart Infusion (BHI) broth were purchased from HiMedia (Mumbai, Maharashtra, India). Dulbecco’s Modified Eagle’s Medium with sodium pyruvate was acquired from Gibco (Grand Island, NY, USA). Gentamicin and MTT were purchased from Bio Basic (Toronto, ON, Canada). Vancomycin, DPPH, ABTS, trolox, and gallic acid were purchased from Sigma-Aldrich (St. Louis, MO, USA). The Folin–Ciocalteu reagent, TPTZ, and quercetin were obtained from Merck (Billerica, MA, USA). Aluminum chloride, ethanol, methanol, potassium acetate, and sodium carbonate were acquired from RCl Labscan (Bangkok, Thailand). Ferrous sulfate, potassium chloride, and sodium acetate were obtained from Qrec (New Zealand). ReverTra Ace^®^ qPCR RT master mix was purchased from TOYOBO (Osaka, Japan). SensiFAST™ SYBR^®^ no-rox kit was purchased from BIOLINE (London, UK). The TUNEL apoptosis assay kit was purchased from abbkine (Atlanta, GA, USA). The TRIzol reagent was acquired from Invitrogen (Carlsbad, CA, USA).

### 2.3. A. platensis Sample and Extraction

**Figure 1:**
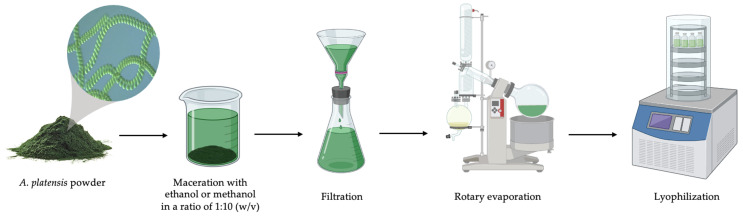
The extraction of *A. platensis* using the maceration method.

The dried powder of *A. platensis* was obtained from Green Diamond Co., Ltd., Chiang Mai Province, Northern Thailand. Moreover, *A. platensis* was characterized using morphology under the compound microscope by Dr. Kritsana Duangjan, Algal and Cyanobacterial Research Laboratory, Department of Biology, Faculty of Science, Chiang Mai University, Chiang Mai, Thailand. The dried powder was extracted through maceration using 95% ethanol and 95% methanol, in a ratio of 1:10 (*w*/*v*). The powder was soaked with solvent for 72 h. Next, the extract mixtures were filtered, concentrated, and freeze-dried using Whatman No. 1 filter paper, a rotary evaporator (Heidolph, Schwabach, Germany), and a lyophilizer (LABCONCO, Kansas City, MO, USA), respectively (Figure 1). The ethanolic and methanolic extracts at a concentration of 250 mg/mL were prepared by dissolving in 99.9% dimethyl sulfoxide (DMSO).

### 2.4. Evaluation of the Antioxidant Activities and Active Compounds

#### 2.4.1. DPPH Radical Scavenging Assay

The measurement of DPPH activity was examined by mixing 150 μL of 0.1 mM DPPH reagent with 50 μL of *A. platensis* extract and incubating in the dark for 20 min. After that, the absorbance was recorded at 517 nm with a microplate reader (DYNEX Technologies, Chantilly, VA, USA). The antioxidant activity was reported as milligrams of gallic acid equivalent per gram of extract (mg GAE/g extract) by comparing to a gallic acid standard curve (1–10 μg/mL) [16].

#### 2.4.2. ABTS Radical Cation Decolorization Assay

The ABTS activity was evaluated by preparing ABTS reagent, which combined 7 mM of ABTS stock solution with 2.45 mM potassium persulfate in a ratio of 1:1 (*v*/*v*) and incubated for 12–16 h. Then, the ABTS reagent was diluted with deionized water to achieve an absorbance of 0.700 ± 0.020 at 734 nm. After that, 195 μL of ABTS reagent was mixed with 5 μL of *A. platensis* extract. Next, the absorbance at 734 nm of the mixture was measured with a microplate reader after incubating in the dark for 10 min. The antioxidant activity was reported as milligrams of Trolox equivalent antioxidant capacity per gram of extract (mg TEAC/g extract) by comparing to a Trolox standard curve (25–450 μg/mL) [17].

#### 2.4.3. Ferric Reducing Antioxidant Power (FRAP) Assay

The FRAP activity of *A. platensis* extracts was determined. FRAP reagent was prepared by a combination of 300 mM acetate buffer (pH 3.6), 10 mM TPTZ in 40 mM HCl, and 20 mM ferric chloride (10:1:1, *v*/*v*/*v*). The 50 μL of *A. platensis* extract was mixed with 100 μL of FRAP reagent. After incubating in the dark for 15 min, the absorbance was read at 593 nm with a microplate reader. The results were reported as mg TEAC/g extract by comparing to a Trolox standard curve (10–100 μg/mL) [16].

#### 2.4.4. Total Phenolic Compound Content

The Folin–Ciocalteau method was used to investigate total phenolic compound content. A 25 μL of *A. platensis* extract was mixed with 12.5 μL of 50% the Folin–Ciocalteu solution, 125 μL of deionized water, and 25 μL of ethanol. After incubating for 5 min, 25 μL of 5% sodium carbonate was added, and then incubated in the dark for 1 h. Next, the absorbance was determined at 725 nm with a microplate reader. Total phenolic compound content was reported as mg GAE/g extract by comparing to a gallic acid standard curve (10–100 μg/mL) [18]. 

#### 2.4.5. Total Flavonoid Compound Content

The colorimetric aluminum chloride method was used to evaluate total flavonoid compound content. The 500 μL of *A. platensis* extract was combined with 100 μL of 10% aluminum chloride, 1.5 mL of methanol, 100 μL of 1 M potassium acetate, and 2.8 mL of deionized water. Then, the mixture was measured for the absorbance at 415 nm with a spectrophotometer (ThermoScientific, Waltham, MA, USA) after incubating in the dark for 30 min. Total flavonoid compound content was reported as milligrams of quercetin equivalent per gram of extract (mg QE/g extract) by comparing to quercetin standard curve (0.98–125 μg/mL) [18].

#### 2.4.6. High Performance Liquid Chromatography Analysis

Gallic acid and quercetin compounds presented in *A. platensis* extracts were identified using the HPLC technique. The ethanolic and methanolic extracts of *A. platensis*, along with standard compounds, were filtrated through a 0.45 μm sterile microfilter. Then, 10 μL of filtered sample was injected into an HPLC system (Agilent Technologies 1260 Infinity II, Santa Clara, CA, USA) equipped with an Agilent Eclipse XDB-C18 column (4.6 × 150 mm, 5 μm). The mobile phase consisted of mobile phase A (0.1% formic acid in water) and mobile phase B (methanol). The gradient elution was set at different ratios of mobile phase A and B (%*v*/*v*) as follows: 50:50 (0 min), 40:60 (20 min), 30:70 (30 min), and 20:80 (40 min). The HPLC condition was operated at 25 °C with a flow rate of 1.0 mL/min. The detection of eluted peaks was carried out at 267 nm, and the amount of compounds in *A. platensis* extracts was calculated by comparing their peak area to standard compounds.

### 2.5. Antibacterial Activities

#### 2.5.1. Bacterial Culture

*S. aureus*, *S. epidermidis*, MRSA, *P. aeruginosa*, and *M. luteus* were cultured on Mueller Hinton agar (MHA) and incubated under aerobic conditions at 37 °C for 18–24 h. *C. acnes* was cultured on Brain Heart Infusion agar (BHIA) and incubated under anaerobic conditions at 37 °C for 48–72 h. 

#### 2.5.2. Agar Well Diffusion Method

The agar well diffusion method was employed to evaluate the antimicrobial susceptibility of *A. platensis* extract against skin pathogenic bacteria. The turbidity of the bacterial suspension was adjusted to achieve an approximate concentration of 1 × 10^8^ CFU/mL, and a sterile cotton swab was swabbed on the agar plates. Then, wells were made by a sterile cork borer and filled with 100 µL of *A. platensis* extract (250 mg/mL). Next, the agar plates were incubated under suitable conditions, and then the inhibition zones were measured in millimeters (mm) in triplicate. A 1 mg/mL concentration of gentamicin and vancomycin was used as positive controls for all bacterial strains and MRSA, respectively. Sterile distilled water and DMSO were used as negative controls [19].

#### 2.5.3. Minimum Inhibitory Concentration (MIC) and Minimum Bactericidal Concentration (MBC)

The determination of MIC was assessed by the broth dilution method. A 100 µL of *A. platensis* extracts (250 mg/mL) and antibiotic positive controls (1 mg/mL) were prepared at different concentrations by two-fold serial dilution and added to a 96-well plate containing sterile broth. Next, 100 µL of bacteria suspension was added and incubated under suitable conditions. After that, the MIC value was examined as the lowest concentration of extract that inhibited visible bacterial growth (no turbidity). The measurement of MBC was evaluated by selecting wells that showed no growth during the MIC determination. After streak plate and incubating under suitable conditions, the MBC value was defined as the lowest concentration of *A. platensis* extract capable of reducing the number of bacterial colonies by up to 99.9% [20].

#### 2.5.4. Inhibition of Biofilm Formation and Biofilm Eradication

Inhibition of biofilm formation and biofilm eradication were determined using the crystal violet (CV) assay with a few modifications. The bacteria were cultured overnight in broth supplemented with 8% glucose. The *A. platensis* extracts and antibiotic positive controls were prepared at sub-minimum bactericidal concentration (1/2 MBC), with the concentration varying depending on the tested bacteria.

Inhibition of biofilm formation was examined by mixing 100 µL of bacterial suspension with 100 µL of each concentration of *A. platensis* extract in a 96-well plate and then incubating without agitation. After that, the reaction mixture was discarded, and wells were gently washed with 1X phosphate-buffered saline (pH 7.4). The 100 µL of 95% ethanol was added for 20 min to fix the adhered cells. Afterwards, the ethanol was discarded, and the well plate was air dried for 20 min. Next, the wells were stained with 120 µL of 0.4% CV solution for 20 min. After removing the unbound dye and washing with 1X PBS, the attached dye was destained with 150 µL of 95% ethanol for 20 min. Then, the 100 µL of the destaining solution was transferred to a new well and measured using a microplate reader at 590 nm. The result was calculated and reported as a percentage of biofilm inhibition by comparing to the control [21,22].

Biofilm eradication was performed by culturing 100 µL of bacteria in a 96-well plate and incubating without agitation to generate the mature biofilm. Subsequently, 100 µL of *A. platensis* extract was added and repeatedly incubated. After that, the wells were removed, washed, fixed, stained, and solubilized as in the inhibition of biofilm formation steps. After completing all steps, the destaining solution was measured using a microplate reader at 590 nm. The result was calculated and reported as a percentage of biofilm eradication by comparing to the control [23].

### 2.6. Anticancer Activity

#### 2.6.1. Cell Culture

The human malignant melanoma cells (A375) (ATCC^®^ CRL-1619™) were purchased from the American Type Culture Collection (Manassas, VA, USA). Cells were cultured in DMEM supplemented with sodium pyruvate, 10% fetal bovine serum, 1% penicillin–streptomycin, and 1% of 1 M HEPES buffer solution. The cell culture was maintained at 37 °C in a 5% CO_2_ incubator.

#### 2.6.2. Cytotoxicity on A375 Cells

The cytotoxicity of *A. platensis* extracts on A375 cells was determined by MTT assay with some modifications. The A375 cells (5 × 10^4^ cells/mL) were cultured in a 96-well plate and incubated for 24 h. Next, the cells were treated with 100 µL of different concentrations of *A. platensis* extracts (0.625–10 mg/mL). After incubating for 48 h, the wells were washed with 1X PBS, and 50 µL of MTT reagent (2 mg/mL in PBS) was added and then incubated for 3 h. The MTT reagent was discarded and filled with 200 µL of DMSO to dissolve the formazan complex for 10 min. The absorbance was recorded with a microplate reader at 540 nm for the tested wavelength and 630 nm for the reference wavelength. Cytotoxicity was calculated and expressed as IC_50_ values when compared with the cell control group [24].

#### 2.6.3. Apoptosis on A375 Cells

Apoptosis in vitro was detected by labeling the DNA fragmentation using the TUNEL apoptosis kit. In brief, A375 cells (at a density of 5 × 10^4^ cells/mL) were cultured in a 96-well plate and incubated for 24 h. Then, the cells were treated with 100 µL of *A. platensis* extracts at the 50% cytotoxicity concentration (IC_50_). After incubating for 48 h, the mixture was discarded and washed with 1X PBS. Next, the cells were fixed with 50 µL of 4% paraformaldehyde, followed by permeabilization with 50 µL of 0.3% Triton X-100 for 30 min. Afterward, the permeabilization solution was removed, and the TUNEL reagent was added and then incubated for 2 h. Thereafter, the nuclei of the cells were stained with DAPI (1:500 in 1X PBS) for 10 min. The apoptotic cells were resuspended in 1X PBS before being observed under a fluorescent microscope and analyzed by flow cytometer [25].

#### 2.6.4. Apoptotic Gene Expression on A375 Cells Using Quantitative Reverse Transcription Polymerase Chain Reaction (RT-qPCR)

A375 cells were treated with *A. platensis* extracts at IC_25_ and IC_50_ doses and incubated for 48 h. Then, the treated cells were washed with 1X PBS, and the total RNA was isolated by a Trizol^®^ reagent in accordance with the manufacturer’s protocol. The isolated RNA was subsequently reverse transcribed into cDNA using ReverTra Ace^®^ qPCR RT Master Mix. The reverse transcription was carried out according to the following protocol: 37 °C for 15 min, 50 °C for 5 min, and 98 °C for 5 min, respectively. Next, qRT-PCR analysis of the synthesized cDNA was performed using SensiFAST™ SYBR^®^ No-ROX Kit, employing specific primers listed in Table 1. Glyceraldehyde-3-phosphate dehydrogenase (GAPDH) was used as the internal control. The qPCR amplification conditions included an initial denaturation at 95 °C for 2 min and 40 cycles of denaturation at 95 °C for 5 s, 65 °C for 30 s in the annealing and extension step, respectively [26].

### 2.7. Statistical Analysis

Statistical analysis was conducted using one-way analysis of variance (ANOVA) with post hoc comparisons performed using Duncan’s multiple range test, all carried out in SPSS Statistic version 26.0. All data are reported as mean ± SD of triplicate experiments. A *p*-value less than 0.05 (*p* < 0.05) was considered to represent a statistically significant difference.

## 3. Results

### 3.1. Antioxidant Activities, Total Phenolic and Flavonoid Compounds of A. platensis Extracts

After the extraction process, the yields of *A. platensis* ethanolic and methanolic extracts were 10.57% and 11.10%, respectively. The ethanolic extract revealed the highest antioxidant activity in DPPH, ABTS, and FRAP assays, with values of 8.96 ± 0.84 mg GAE/g extract, 53.03 ± 4.21 mg TEAC/g extract, and 48.06 ± 0.78 mg TEAC/g extract, respectively (Table 2).

Moreover, the ethanolic extract of *A. platensis* contained higher levels of total phenolic and flavonoid compound contents than the methanolic extract, with values of 38.79 ± 1.61 mg GAE/g extract and 27.50 ± 0.53 mg QE/g extract, respectively (Table 3).

### 3.2. Identification and Quantification of Bioactive Compounds in A. platensis Extracts

**Figure 2:**
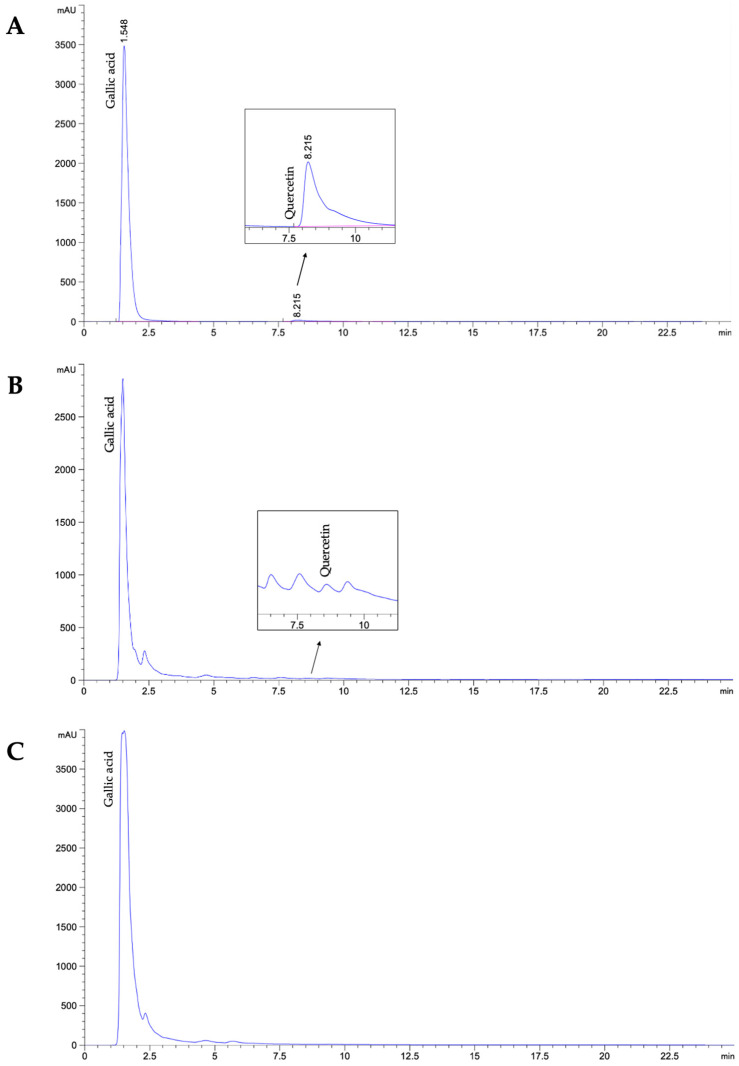
HPLC chromatogram of standard mixture of gallic acid and quercetin (**A**), ethanolic (**B**), and methanolic (**C**) extracts of *A. platensis*.

The bioactive compounds, including gallic acid and quercetin, in the ethanolic and methanolic extracts of *A. platensis* were analyzed using the HPLC technique. Gallic acid was present in both extracts, with values of 20.50 ± 0.03 mg/g extract in the ethanolic extract and 21.84 ± 0.77 mg/g extract in the methanolic extract. Quercetin was detected only in the ethanolic extract, with a value of 0.09 ± 0.01 mg/g extract (Table 4 and Figure 2).

### 3.3. Antibacterial Activities of A. platensis Extracts

The antibacterial activity of *A. platensis* extracts at a concentration of 250 mg/mL was tested against skin pathogenic bacteria, including *Staphylococcus aureus*, *Staphylococcus epidermidis*, methicillin-resistant *S. aureus* (MRSA), *Micrococcus luteus*, *Pseudomonas aeruginosa*, and *Cutibacterium acnes* using agar well diffusion method. The ethanolic extract of *A. platensis* demonstrated superior antibacterial activity compared to the methanolic extract. Moreover, the ethanolic extract inhibited all skin pathogenic bacteria, except MRSA and *P. aeruginosa*, with the inhibition zone ranging from 9.67 ± 0.58 to 12.50 ± 0.50 mm. Gentamicin and vancomycin, which were used as positive controls, exhibited the inhibition zone ranging from 28.33 ± 0.76 to 37.50 ± 2.18 mm, while the negative control (DMSO) displayed no inhibition zone (Table 5).

### 3.4. Minimum Inhibitory Concentration (MIC) and Minimum Bactericidal Concentration (MBC) of A. platensis Extracts

The MIC and MBC determination of *A. platensis* extracts at a concentration of 250 mg/mL were evaluated using the broth dilution method. The results illustrated that the ethanolic and methanolic extracts of *A. platensis* were against all skin pathogenic bacteria, with MIC and MBC values in the range of 31.25 to 125 mg/mL (Table 6). Gentamicin and vancomycin at a concentration of 1 mg/mL, used as a positive control for all tested bacteria, including MRSA, inhibited bacterial growth, with MIC and MBC values between 0.0039 and 0.0625 mg/mL.

### 3.5. Inhibition of Biofilm Formation and Biofilm Eradication of A. platensis Extracts

**Figure 3:**
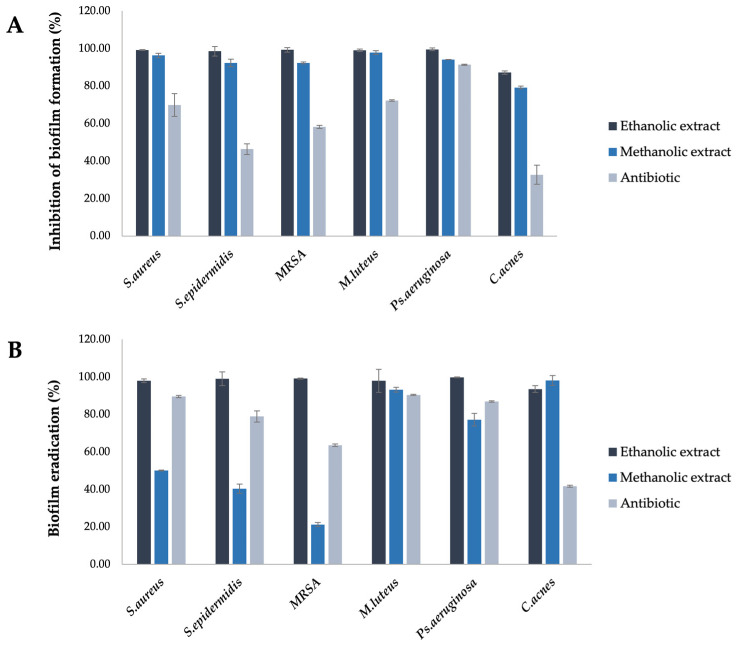
The inhibition of biofilm formation (**A**) and biofilm eradication (**B**) of *A. platensis* extracts against skin pathogenic bacteria using the crystal violet staining method.

The inhibition of biofilm formation and biofilm eradication of *A. platensis* extracts at sub-MBC (62.5 mg/mL) was assessed by the crystal violet staining method. The ethanolic extract of *A. platensis* showed the inhibition of biofilm formation rates ranging from 87.18% to 99.53%, and biofilm eradication rates from 93.51% to 99.77%. The methanolic extract of *A. platensis* displayed the inhibition of biofilm formation and eradication rates ranging from 79.15% to 97.87% and 21.29% to 98.13%, respectively. Thus, the ethanolic extract of *A. platensis* demonstrated greater efficacy in inhibiting and eradicating biofilm than the methanolic extract. The methanolic extract was the most effective extract for eradicating *C. acnes* biofilm, while it had the least impact on MRSA biofilm. Antibiotics used as positive controls exhibited the inhibition of biofilm formation and eradication with rates ranging from 32.64% to 91.33% and 41.74% to 90.39%, respectively. The antibiotics were the least effective against *C. acnes* biofilm in both biofilm inhibition and eradication assays (Figure 3).

### 3.6. Cytotoxicity of A. platensis Extracts in A375 Cells

**Figure 4:**
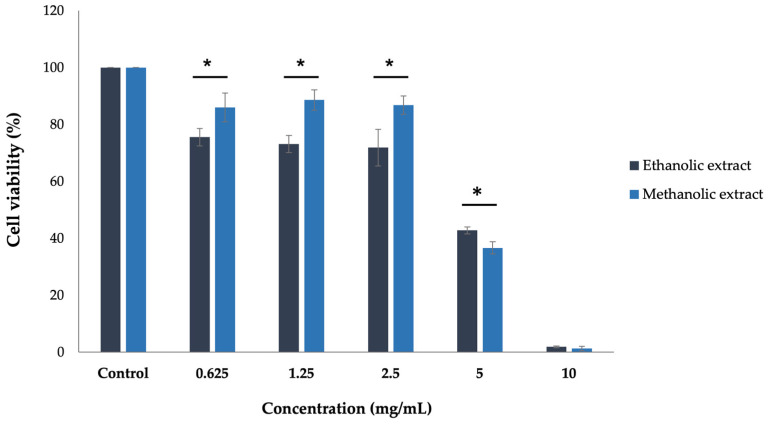
Effect of ethanolic and methanolic extracts of *A. platensis* on the viability of A375 human melanoma cells. Cells were treated with various concentrations (0.625–10 mg/mL) for 48 h, and cell toxicity was assessed by MTT assay. * Indicates a statistically significant difference (*p* < 0.05) compared to the cell treated with ethanolic and methanolic extract.

The cytotoxic effect of *A. platensis* extracts in A375 cells was performed after treatment with ethanolic and methanolic extracts at concentrations of 0.625, 1.25, 2.5, 5, and 10 mg/mL for 48 h. The MTT assay was used to evaluate cell viability compared to the untreated control cells. The ethanolic extract exhibited a 50% inhibitory concentration (IC_50_) of 4.42 ± 0.56 mg/mL, while the methanolic extract had an IC_50_ of 5.31 ± 0.59 mg/mL. This finding indicated that the ethanolic extract of *A. platensis* is more effective in inhibiting the growth of A375 human melanoma cells than the methanolic extract. Therefore, the IC_50_ values were selected for use in subsequent experiments (Figure 4).

### 3.7. Effect of A. platensis Extracts on Apoptosis in A375 Cells

**Figure 5:**
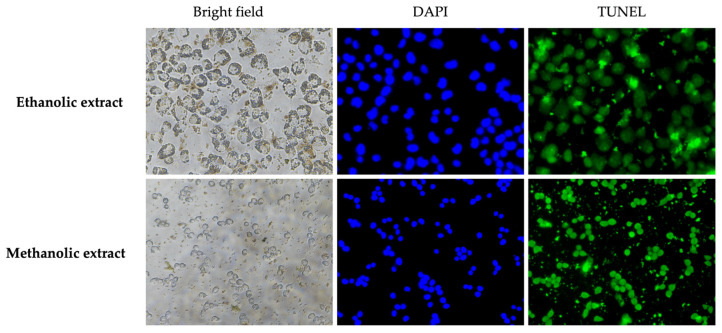
Apoptosis of A375 human melanoma cells treated with ethanolic and methanolic extracts of *A. platensis* at an IC_50_ dose. Apoptotic cells were detected using the TUNEL assay and visualized under a fluorescent microscope.

**Figure 6:**
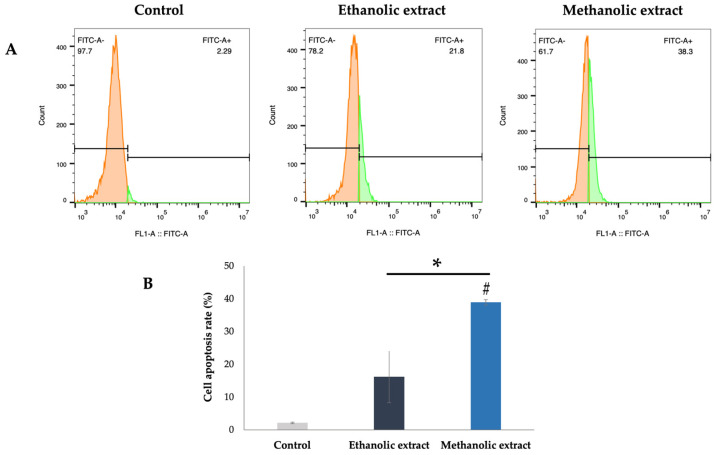
Quantification of cell apoptosis in A375 human melanoma cells treated with ethanolic and methanolic extracts of *A. platensis* at an IC_50_ dose and detected by flow cytometry (**A**). # Indicates a statistically significant difference (*p* < 0.05) compared to control cells. * Indicates a statistically significant difference (*p* < 0.05) compared to cells treated with ethanolic and methanolic extracts (**B**).

The apoptosis mechanisms of *A. platensis* extracts were studied by inducing the DNA fragmentation in A375 cells using the TUNEL assay, followed by detection with a fluorescent microscope and flow cytometry. The results indicated that the positive cells, labeled with TUNEL green fluorescent, were observed after treatment with ethanolic and methanolic extracts at 50% inhibitory concentration (IC_50_) (Figure 5). The results demonstrated that both ethanolic and methanolic extracts of *A. platensis* were able to trigger cell apoptosis in A375 cells compared to the untreated control cells. Furthermore, the methanolic extract showed greater induction of DNA fragmentation than the ethanolic extract, as evidenced by flow cytometry. The apoptosis rates for the ethanolic and methanolic extracts were 16.20 ± 7.92% and 38.90 ± 0.85%, respectively, compared to the untreated control cells (Figure 6).

### 3.8. Effect of A. platensis Extracts on Apoptotic Gene Expression in A375 Cells 

**Figure 7:**
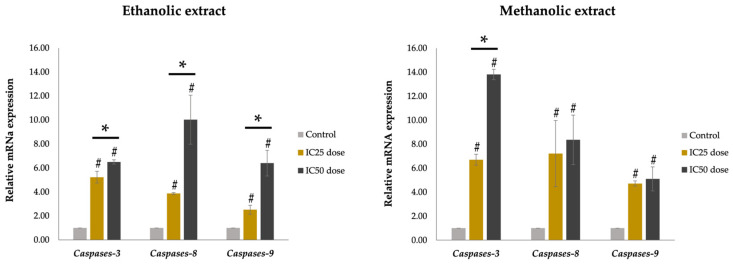
RT-qPCR analysis of apoptosis gene expression levels of *Caspase-3*, *Caspase-8*, and *Caspase-9* after A375 human melanoma cells were treated with ethanolic and methanolic extracts of *A. platensis* at IC_25_ and IC_50_ doses. # Indicates a statistically significant difference (*p* < 0.05) compared to untreated control cells. * Indicates a statistically significant difference (*p* < 0.05) compared to cells treated with extracts at IC_25_ and IC_50_ doses.

The effect of *A. platensis* extract on the apoptotic gene expression in A375 cells was performed after treatment with ethanolic and methanolic extracts at IC_25_ and IC_50_ doses. After 48 h of incubation with the extract, the relative mRNA expression levels of *Caspase-3*, *Caspase-8*, and *Caspase-9* were analyzed by RT-qPCR technique. The results revealed that both ethanolic and methanolic extracts increased apoptotic gene expression in a concentration-dependent manner. Both extracts upregulated the expression of *Caspase-3*, *Caspase-8*, and *Caspase-9* genes compared to the untreated control group. The ethanolic and methanolic extracts of *A. platensis* induced cell apoptosis via both extrinsic and intrinsic apoptosis pathways, which are related to the induction of *Caspase-8* and *Caspase-9*, respectively. The methanolic extract showed a greater ability to trigger cell apoptosis than the ethanolic extract (Figure 7).

## 4. Discussion

*Arthrospira platensis* (spirulina) contains a variety of micro- and macronutrients that are essential for basic human nutrition. Moreover, it is rich in bioactive compounds such as fatty acids, phycocyanin, carotenoids, chlorophyll, polysaccharides, and polyphenols that are associated with antioxidant, antimicrobial, antiviral, anti-inflammatory, anticancer, and other beneficial properties [27,28]. The bioactive compounds of *A. platensis* extracts were analyzed using TLC, GC-MS, and HPLC-UV techniques. The analysis reported that the ethanolic extract of *A. platensis* contained bioactive compounds including p-coumaric acid, caffeic acid, chlorogenic acid, ferulic acid, syringic acid, apigenin, kaempferol, and quercetin [17]. Furthermore, the bioactive components identified in *A. platensis* methanolic extract included hexadecanoic acid, 9-octadecanoic acid, tetradecanoic acid, hexadecanal, and phytol [29,30]. Previous studies analyzed the phytochemical constituents of *A. platensis* extracts prepared using ethanolic and methanolic solvents, revealing various biochemical compounds, including alkaloids, glycosides, flavonoids, phenols, terpenoids, steroids, and saponins [31]. This study determined the total phenolic and flavonoid compounds, indicating that *A. platensis* ethanolic extract exhibited high levels of these compounds and exhibited notable antioxidant activity in DPPH, ABTS, and FRAP assays.

Moreover, the HPLC analysis revealed that the ethanolic extracts of *A. platensis* also contained gallic acid and quercetin at concentrations of 20.50 ± 0.03 mg/g extract and 0.09 ± 0.01 mg/g extract, respectively. The presence of gallic acid in the *A. platensis* may be associated with the total phenolic compound content, as it is one of the phenolic derivatives. Previous studies have also detected phenolic compounds, including caffeic acid, chlorogenic acid, coumaric acid, and gallic acid in *A. platensis* extract [32]. However, the gallic acid content reported in this study was higher than that previously observed. Conversely, the flavonoid compound content in this study was relatively low, with quercetin detected in minimal amounts. Since quercetin was the only standard compound used for flavonoid analysis in this study, it may not represent the total flavonoid compound content since *A. platensis* contains a variety of flavonoid derivatives, apart from quercetin. There are several studies that have exhibited the flavonoid compounds present in *A. platensis*, including apigenin, catechin, epicatechin, kaempferol, naringenin, naringin, and rutin [33,34].

The strong antioxidant properties of phenolic and flavonoid compounds can be attributed to their multiple hydroxyl groups on aromatic rings, which effectively act as hydrogen donors, neutralizing reactive oxygen species [35,36]. Total phenolic and flavonoid compound contents are directly linked to antioxidant activity, with higher levels in *A. platensis* extract resulting in greater free radical scavenging activity [37]. Additionally, it is reported that these bioactive compounds derived from *A. platensis* exert antioxidant effects through multiple synergistic mechanisms, including scavenging of reactive oxygen species (ROS) such as hydroxyl radicals, singlet oxygen, and superoxide, chelating metal ions, donating hydrogen and electron atoms, and inhibiting membrane lipid peroxidation [38]. Nevertheless, the total phenolic and flavonoid compound contents reported in this study were inconsistent with earlier research [39]. These variations may be attributed to differences in various factors such as sample origin, sample cultivation (e.g., temperature, humidity, nutrients, light, etc.), solvents used, material-to-solvent ratio, extraction techniques, and extraction time [31,40].

As *A. platensis* contains many bioactive compounds, it has a broad-spectrum antimicrobial activity against pathogenic microorganisms, including bacteria, fungi, yeasts, and viruses. In our study, *A. platensis* extracts were investigated for their antibacterial activity against skin pathogenic bacteria by agar well diffusion and broth dilution methods. Gentamicin and vancomycin were used as a positive control due to their broad-spectrum antimicrobial properties. Gentamicin acts by disrupting bacterial protein synthesis, while vancomycin inhibits bacterial cell wall formation [41,42]. The results demonstrated that the ethanolic extract of *A. platensis* inhibited all tested bacteria, except MRSA and *P. aeruginosa*. In contrast, the methanolic extract of *A. platensis* did not show activity against *S. epidermidis* using the agar well diffusion method. Nevertheless, both ethanolic and methanolic extracts of *A. platensis* revealed antibacterial activity against all tested bacteria in the broth dilution method, with MIC and MBC values in the range of 31.25 mg/mL to 125 mg/mL.

The *A. platensis* extracts demonstrated the potential antibacterial activity at higher concentrations. This effect might be due to the presence of complex compositions in the crude extract, which interact with some bioactive compounds and could lead to reducing individual effects and result in diminished overall efficacy against bacterial cells. However, research on the inhibitory effect of *A. platensis* ethanolic extracts against dermatophytes isolated from patients reported a notable effect against *Trichophyton rubrum*, *Trichophyton concentricum*, and *Trichophyton interdigitale* when using the *A. platensis* extracts at 100 and 200 mg. This research indicated that the antimicrobial activity of the extract was increased proportionally when using higher concentrations [43].

These findings indicated that although the ethanolic and methanolic *A. platensis* extracts did not show the inhibition zone against some bacteria using agar well diffusion method, both extracts still demonstrated antibacterial effects when determined by the broth dilution method. This could be attributed to the diffusion ability of the extract through the agar. As noted by Hossain (2024), factors such as molecular weight, solubility, and diffusion rate of the chemical compound in the extract influence its diffusion on agar [44]. However, the broth dilution methods offer certain advantages over the agar diffusion method by enabling direct contact between the extracts and bacterial cells, resulting in greater effectiveness. This finding indicated that Gram-positive bacteria were more susceptible to *A. platensis* extract than Gram-negative bacteria, consistent with previous studies [45], which have reported that the *A. platensis* extract is more effective in inhibiting the growth of Gram-positive bacteria. This difference is likely due to the complicated structure of the outer membrane in the cell wall of Gram-negative bacteria [46].

Biofilm is a complex community of bacteria enclosed in an extracellular polymeric substance matrix, consisting of polysaccharides, proteins, and extracellular DNA, which provides strong adhesion and persistent properties [47,48]. Biofilm formation plays an important role in enhancing bacterial growth and increasing resistance to treatments, posing a significant challenge in the treatment of bacterial infections. Therefore, the natural extract with diverse biochemical compositions provides a promising alternative treatment for the management of pathological conditions. Our study evaluated the ability of *A. platensis* extract to inhibit biofilm formation and biofilm eradication. The results showed that both ethanolic and methanolic extracts of *A. platensis*, at sub-MBC, effectively inhibited biofilm formation and eliminated the mature biofilm in all tested bacteria, with inhibitory efficacy ranging from 21.29% to 99.77%. Previous studies have reported similar biofilm inhibition by *A. platensis* methanolic extract against a wide range of clinical Gram-positive and Gram-negative bacteria, with inhibition rates ranging from 49% to 90%. Moreover, *A. platensis* extract has been shown to reduce exopolysaccharide production and cell surface hydrophobicity (CSH), which are related to biofilm formation [48]. According to Mirani et al. (2018), cell surface hydrophobicity is a crucial factor in promoting bacterial attachment and the development of biofilm [49]. Similarly, earlier studies have shown a strong correlation between cell surface hydrophobicity and biofilm formation. Increased hydrophobicity levels facilitate the development of multicellular aggregates of cell consortia, which exhibit strong adhesion to the surface, resulting in the robust biofilm formation that is long-lasting and difficult to disperse [50]. Other studies indicated that the reduction in cell surface hydrophobicity of *P. aeruginosa* leads to a decrease in bacterial cell adhesion [51].

*A. platensis* extracts and their bioactive compounds have been evaluated for cytotoxicity on different cell types, such as T47D, P388, K562, Kasumi-1, Caco-2, HepG2, MCF7, and L20B cells [30,52,53,54]. This study specifically investigates the cytotoxicity of *A. platensis* extracts on A375 human melanoma cells. The results demonstrated that both ethanolic and methanolic extracts of *A. platensis* showed significant toxicity toward A375 human melanoma cells, with IC_50_ values of 4.42 and 5.31 mg/mL, respectively. Consistent with previous research, the ethanolic extract of *A. platensis* has been shown to significantly reduce cell viability in colon cancer (Caco-2) and liver cancer (HepG2) cells in a concentration-dependent manner, since cell viability decreased when increasing extract concentrations. Importantly, no adverse effects were observed on the normal human fibroblast cell line (HdFn) [54]. Fayyad et al. (2019) presented the cytotoxic effect of *A. platensis* methanolic extract against human cancer cells, including MCF7 and L20B cells, showing a concentration- and time-dependent manner compared to control cells [30]. Similarly, Akbarizare et al. (2020) reported the cytotoxic effects of *A. platensis* crude extract and its secondary metabolites on HepG2 cells [55]. The results revealed that crude extract and isolated secondary metabolites of *A. platensis* inhibited the proliferation of HepG2 cells in a concentration-dependent manner, without impacting the viability of normal fibroblast cells. Moreover, two major secondary metabolites, phenolic and alkaloid compounds, exhibited cytotoxic effects approximately 2.5-fold to 3-fold greater than the *A. platensis* crude extract. This enhanced activity could be attributed to a complex mixture of substances in the *A. platensis* crude extract, where some bioactive compounds may exhibit lower potency in their combined form. Furthermore, *A. platensis* has been reported not only for its non-toxic effects on normal cells but also for its ability to stimulate the proliferation of various human dermal skin cell types, including human fibroblast (HFF1), epidermal fibroblast (HFFF-2), dermal fibroblast (CCD-986sk), and keratinocyte (HaCaT) cells. This proliferative effect highlights its potential role in enhancing wound healing properties and tissue regeneration [56,57].

The anticancer activity was further observed by the determination of apoptosis in the A375 cancer cells using TUNEL green fluorescence, which was observed through a fluorescence microscope and analyzed by flow cytometry. Apoptosis was confirmed by the upregulation of caspase gene expression. Caspases are a group of cysteine proteases that regulate apoptosis, or cell death, through two signaling pathways, the extrinsic and intrinsic pathways. The extrinsic pathway is triggered by extracellular signals that are recognized and transmitted through interaction with death receptors, resulting in activation of caspase-8. In contrast, the intrinsic pathway is driven by mitochondrion-centered processes that are characterized by mitochondrial outer membrane permeabilization (MOMP), which leads to the formation of the apoptosome and the stimulation of caspase-9. Both pathways can directly activate apoptosis through caspase-3 and caspase-7 [58,59]. This study evaluated the apoptotic gene expression of *Caspase-3*, *Caspase-8*, and *Caspase-9* in response to *A. platensis* extract treatment by RT-qPCR. The results were presented as relative mRNA expression levels. Both ethanolic and methanolic extracts of *A. platensis* at IC_50_ doses induced apoptosis in A375 human melanoma cells by 16.20% and 38.90%, respectively. Furthermore, the *A. platensis* extracts stimulate apoptosis through extrinsic and intrinsic pathways by upregulating *Caspase-3*, *Caspase-8*, and *Caspase-9* expression in a dose-dependent manner, compared to the untreated control group.

The cytotoxic and apoptotic effects of *A. platensis* extracts may be attributed to its diverse phytochemical constituents, as supported by previous research. Flavonoids exhibited various effects on cancer cells, including inhibiting cell proliferation and differentiation, suppressing kinase enzyme activity, triggering cell cycle arrest, and inducing apoptosis [60]. Quercetin could induce apoptosis via both intrinsic and extrinsic pathways by upregulating the expression of caspase-3, caspase-8, and caspase-9 in cancer cells. Furthermore, it reduced survival signaling by decreasing the expression of Bcl-2, Mcl-1, and Bcl-xl, and altering the Bcl-xL: Bcl-xS ratio [61,62]. Phenolic compounds derived from *A. platensis* have demonstrated pro-apoptotic effects against esophageal cancer cells (SK-GT-4) by inducing apoptosis [63]. Saponins have been reported to suppress cell proliferation and promote apoptosis through caspase-3 activation in human colon cancer cells (HT-29) and tumor xenograft models [64]. Terpenoids and their derivatives possess numerous medicinal properties, including immunomodulation, antiallergenic, antiparasitic, anticancer, and anti-inflammatory activities. Certain terpenoids exhibit antitumor effects by targeting different stages of cancer progression. During the initiation stage of tumorigenesis, they induce cell cycle arrest, inhibit cell differentiation, and promote apoptotic cell death, while in later stages, they suppress angiogenesis and metastasis by regulating various intracellular signaling pathways [65]. Furthermore, *A. platensis*-derived phycocyanin has shown significant pro-apoptotic effects on cancer cells by increasing the expression of caspase-3, caspase-8, and caspase-9, activating the p53 gene, and promoting cytochrome *c* release [66]. Its anticancer effects are also associated with mitochondrial dysfunction through the upregulation of pro-apoptotic Bax protein expression and the downregulation of anti-apoptotic Bcl-2 protein expression [67]. In addition, several fatty acids from the *A. platensis* extract, especially polyunsaturated fatty acids (PUFAs), have anticancer effects through different pathways, including enhancement of reactive oxygen species (ROS) production, stimulation of caspase-dependent apoptotic pathways, accumulation of cytotoxic lipid peroxidation byproducts that promote apoptosis, activation of peroxisome proliferator-activated receptors (PPARs), modulation of oncogene and tumor suppressor gene expression, and interaction with chromosomal components of cancer cells [38,68].

**Figure 8:**
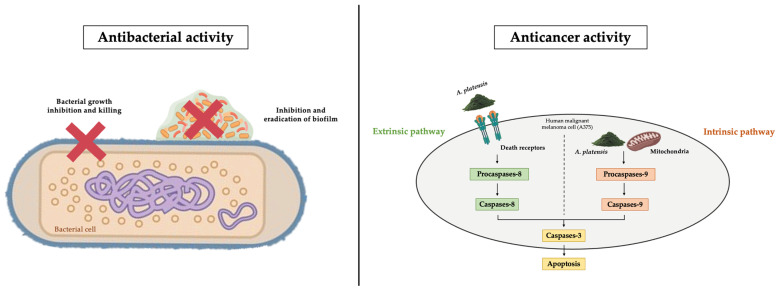
The mechanisms of antibacterial and anticancer activities of *A. platensis* extracts.

In summary, the results demonstrated that *A. platensis* contains diverse bioactive compounds with potential antibacterial and anticancer properties, with their predicted mechanisms of action presented in Figure 8. Nevertheless, this study has certain limitations. Although the antioxidant, antibacterial, and anticancer activities of ethaniolic and methanolic extracts of *A. platensis* were demonstrated, the complexity of crude mixtures makes it challenging to pinpoint the specific active components. Therefore, the further identification of individual bioactive compounds from *A. platensis* extract should be performed. Moreover, the assessment of anticancer effects on other cancer cells, as well as *in vivo* validation, is still necessary to fully evaluate the potential of *A. platensis* extracts for future therapeutic applications in many diseases.

## 5. Conclusions

This study demonstrated that *A. platensis* extracts exhibit potent antioxidant activity, which may be attributed to their total phenolic and flavonoid compound contents. Moreover, the antibacterial activity against skin pathogenic bacteria, including *S. aureus*, *S. epidermidis*, methicillin-resistant *S. aureus* (MRSA), *M. luteus*, *P. aeruginosa*, and *C. acnes*, showed a greater inhibitory effect against Gram-positive bacteria compared to Gram-negative bacteria because of the differences in their cell wall structures. Additionally, the *A. platensis* extract displayed cytotoxicity to A375 human melanoma cells and induced apoptosis via both intrinsic and extrinsic pathways. Overall, this study indicates that *A. platensis* extracts could be beneficial as an alternative therapeutic option for treating bacterial infections, free radicals, and cancer cells. However, further investigation into its clinical and pharmaceutical applications is necessary for future product development.

## Figures and Tables

**Table 1:** Specific primers used in RT-qPCR amplification.

Genes	Forward Primer Sequences 5′-3′	Reverse Primer Sequences 3′-5′
*Caspase-3*	TGTTTGTGTGCTTCTGAGCC	TCAAGCTTGTCGGCATACTG
*Caspase-8*	GTGGAGGAAAGCAATCTGTC	TATTAGCCCTGCCTGGTGTCT
*Caspase-9*	GACTCCCTCGAGTCTCCAGAT	GACTCCCTCGAGTCTCCAGAT
GAPDH	GAAGGTGAAGGTCGGAGTC	GAAGATGGTGATGGGATTTC

**Table 2:** Antioxidant activities of *A. platensis* extracts.

*A. platensis*	DPPH (mg GAE/g Extract)	ABTS (mg TEAC/g Extract)	FRAP (mg TEAC/g Extract)
Ethanolic extract	8.96 ± 0.84 ^a^	53.03 ± 4.21 ^a^	48.06 ± 0.78 ^a^
Methanolic extract	3.50 ± 0.23 ^b^	43.44 ± 2.36 ^b^	19.70 ± 0.38 ^b^

The data in this table is represented as mean ± SD, different subscript letters (^a, b^) indicate a statistically significant difference (*p* < 0.05).

**Table 3:** Total phenolic and flavonoid compound contents in *A. platensis* extracts.

*A. platensis*	Total Phenolic (mg GAE/g Extract)	Total Flavonoid (mg QE/g Extract)
Ethanolic extract	38.79 ± 1.61 ^a^	27.50 ± 0.53 ^a^
Methanolic extract	23.71 ± 0.93 ^b^	25.85 ± 0.51 ^b^

The data in this table is represented as mean ± SD, different subscript letters (^a, b^) indicate a statistically significant difference (*p* < 0.05).

**Table 4:** Bioactive compounds in *A. platensis*.

*A. platensis*	Gallic Acid (mg/g Extract)	Quercetin Extracts Identified by HPLC (mg/g Extract)
Ethanolic extract	20.50 ± 0.03 ^a^	0.09 ± 0.01
Methanolic extract	21.84 ± 0.77 ^a^	Undetected

The data in this table is represented as mean ± SD. The same subscript letter (^a^) indicates no statistically significant difference (*p* < 0.05).

**Table 5:** The antibacterial effects of *A. platensis* extracts on skin pathogenic bacteria.

*A. platensis*	Inhibition Zone Diameter (mm)					
	Skin Pathogenic Bacteria					
	*S. aureus*	*S. epidermidis*	MRSA	*M. luteus*	*P. aeruginosa*	*C. acnes*
Ethanolic extract	11.33 ± 1.04 ^bc^	12.40 ± 0.96 ^c^	0 ^a^	12.50 ± 0.50 ^c^	0 ^a^	9.67 ± 0.58 ^b^
Methanolic extract	10.77 ± 0.25 ^bc^	0 ^a^	0 ^a^	10.83 ± 0.58 ^bc^	0 ^a^	11.00 ± 0.50 ^bc^
Gentamicin (1 mg/mL)	28.33 ± 0.76 ^d^	29.50 ± 1.73 ^d^	ND	37.50 ± 2.18 ^g^	32.17 ± 0.58 ^e^	35.67 ± 1.89 ^f^
Vancomycin (1 mg/mL)	ND	ND	28.67 ± 1.04 ^d^	ND	ND	ND
DMSO	0 ^a^	0 ^a^	0 ^a^	0 ^a^	0 ^a^	0 ^a^

The data in this table is represented as mean ± SD; different subscript letters (^a–g^) indicate a statistically significant difference (*p* < 0.05). ND: Not determined.

**Table 6:** The MIC and MBC values of *A. platensis* extracts against skin pathogenic bacteria.

*A. platensis*	MIC and MBC (mg/mL)											
	Skin Pathogenic Bacteria											
	*S. aureus*		*S. epidermidis*		MRSA		*M. luteus*		*P. aeruginosa*		*C. acnes*	
	MIC	MBC	MIC	MBC	MIC	MBC	MIC	MBC	MIC	MBC	MIC	MBC
Ethanolic extract	125	125	125	125	31.25	125	31.25	125	125	125	125	125
Methanolic extract	125	125	62.5	125	125	125	125	125	125	125	125	125
Gentamicin (1 mg/mL)	0.0039	0.0039	0.0156	0.0156	ND	ND	0.0039	0.0039	0.0039	0.0039	0.0625	0.0625
Vancomycin (1 mg/mL)	ND	ND	ND	ND	0.0312	0.0312	ND	ND	ND	ND	ND	ND

ND: Not determined.

## Data Availability

The original contributions presented in this study are included in the article. Further inquiries can be directed to the corresponding author.

## Abbreviations

The following abbreviations are used in this manuscript:

ABTS	2, 2-azinobis (3- ethylbenzothiazoline-6 -sulfonic acid)
DMEM	Dulbecco’s modified Eagle’s medium
DPPH	2, 2-diphenyl-1-picrylhydrazyl
FRAP	Ferric reducing antioxidant power
GAE	Gallic acid equivalent
GAPDH	Glyceraldehyde-3-phosphate dehydrogenase
HPLC	High-performance liquid chromatography
MRSA	Methicillin-resistant *S. aureus*
MTT	3-(4,5-dimethylthizaol-2-yl)-2,5-diphenyl tetrazolium bromide
QE	Quercetin equivalent
RT-qPCR	Quantitative reverse transcription polymerase chain reaction
TEAC	Trolox equivalent antioxidant capacity
TPTZ	2,4,6-tri(2-pyridyl)-s-triazine
Trolox	6-hydroxy-2,5,7,8-tetramethyl-chroman-2-carboxylic acid
TUNEL	Terminal deoxynucleotidyl transferase dUTP nick-end labeling
